# Speed change discrimination for motion in depth using constant world and retinal speeds

**DOI:** 10.1371/journal.pone.0214766

**Published:** 2019-04-03

**Authors:** Abigail R. I. Lee, Justin M. Ales, Julie M. Harris

**Affiliations:** School of Psychology and Neuroscience, University of St Andrews, St Andrews, Fife, United Kingdom; Universite Toulouse III - Paul Sabatier, FRANCE

## Abstract

Motion at constant speed in the world maps into retinal motion very differently for lateral motion and motion in depth. The former is close to linear, for the latter, constant speed objects accelerate on the retina as they approach. Motion in depth is frequently studied using speeds that are constant on the retina, and are thus not consistent with real-world constant motion. Our aim here was to test whether this matters: are we more sensitive to real-world motion? We measured speed change discrimination for objects undergoing accelerating retinal motion in depth (consistent with constant real-world speed), and constant retinal motion in depth (consistent with real-world deceleration). Our stimuli contained both looming and binocular disparity cues to motion in depth. We used a speed change discrimination task to obtain thresholds for conditions with and without binocular and looming motion in depth cues. We found that speed change discrimination thresholds were similar for accelerating retinal speed and constant retinal speed and were notably poor compared to classic speed discrimination thresholds. We conclude that the ecologically valid retinal acceleration in our stimuli neither helps, nor hinders, our ability to make judgements in a speed change discrimination task.

## Introduction

The perception of motion is crucial for human vision: not only do we live in a world where important objects move, but we also move ourselves. Human perception of the motion around us has been studied in great detail. From this we know our visual system performs specific processing of motion speed and direction (for a recent review, see [1]). Because the retina can be thought of as a two-dimensional array, the vast majority of this work has been on motion constrained to the two dimensions that specify the fronto-parallel plane. In the world, however, motion actually occurs in three dimensions. It is therefore essential to understand motion in both two and three dimensions, and to understand the differences between them.

### Differences between 2D and 3D motion

**Fig 1:**
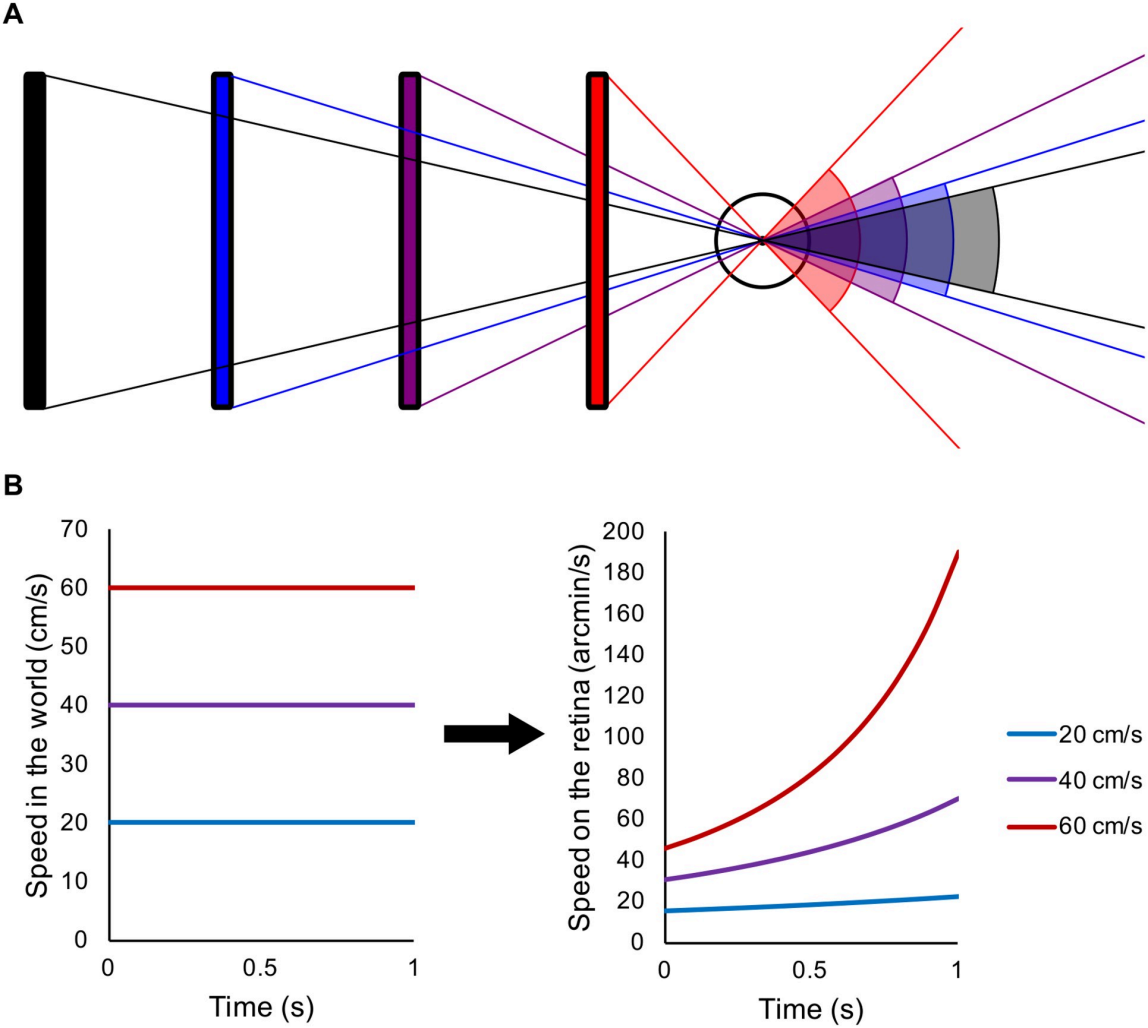
How constant world speed is translated into accelerating retinal speed. (A) Illustrates how objects moving at a constant speed in depth in the world results in acceleration on the retina. Black, blue, purple and red lines/angles respectively, represent the object location/retinal projection at 4 consecutive time points. (B) Constant speed in the world (left) maps to accelerating speed on the retina (right). From a starting position of 117cm from the eye, objects travelling towards the eye at three different speeds accelerate, increasing rapidly in speed the closer to the eye the objects get.

There are two key differences between 2D and 3D motion: (i) the mapping between speed in the world and on the retina, and (ii) the visual cues available for each. For 2D motion, speed in the world is proportional to speed on the retina, with the exact proportion depending on distance to the observer. When an object undergoes fairly small motions in the fronto-parallel plane (lateral motion), the distance to the observer stays relatively constant, therefore a constant speed in the world results in an effectively constant speed on the retina. However, when an object changes distance from the observer, this relationship breaks down. When an object moves towards or away from an observer at a constant speed in the real world, the image of that object on the retina accelerates or decelerates respectively, as shown in Fig 1A, and described in a recent review [2]. Fig 1A depicts 4 bars, each corresponding to the same object in the world at different distances from the retina, with the visual angle subtended by each bar’s retinal image in the right panel. The red bar, closest to the eye, subtends the largest retinal image. The black bar that is the furthest away from the eye creates the smallest retinal image. Whilst the distance between each bar is the same in the world, as a bar moves closer to the eye at a constant world speed, the distance moved between two locations in depth causes a greater change in visual angle, generating retinal acceleration as an object approaches the observer. This acceleration on the retina is not constant: it increases as the object approaches the observer. Many experiments on motion in depth have used constant retinal speed (for example, [3–9]). Such motion is consistent with the stimulus decelerating in the world. To represent a constant speed in the world, retinal motion must accelerate as an object approaches (see Fig 1B).

For lateral motion, the major cue used to determine speed is the retinal speed of motion. Speed discrimination has been studied extensively for 2D lateral motion, and within an optimal range of speeds, people can discriminate between speeds that are only 5–7% different [10–15].

For motion in depth, there are both binocular and monocular cues available. There are two distinct binocular cues to motion in depth: changing disparity (CD) and interocular velocity difference (IOVD; see [16] for a review). Even with both cues available, speed discrimination is significantly worse than the best possible thresholds for lateral motion [3,4,17].

The major monocular cue for motion in depth is looming, where the retinal image of an object moving at a constant speed in the world expands at an accelerating rate as the object approaches the observer (e.g. see Fig 1A). Speed discrimination thresholds of around 5% have been reported for looming motion [18]. Similarly, speed discrimination thresholds of around 7% have been reported using expanding optic flow fields [19].

### Use of constant world and retinal speeds

There is a complication in the literature around the perception of motion in depth, namely that some studies have used constant world motion (e.g. [18,20,21]), and some have used constant retinal motion (e.g. [3–9]). This is potentially a problem because if there is a difference in how we discriminate the speed of motion in depth with a constant world speed or a constant retinal speed, we may have been making assumptions about motion in depth that are not appropriate for more ecologically valid stimuli.

Many experiments conducted to investigate the perception of motion in depth have used constant retinal speeds (e.g. [3–8]). Constant retinal speeds were also used in one of the few examples of where looming and binocular cues to motion in depth have been studied together [9].

Studies that have used accelerating retinal speed with motion in depth are less common. Examples using looming include Sekuler’s [18] speed discrimination study. Another study including looming, changing disparity and vergence information used accelerating retinal speed and reported that these cues contribute differently to our perception of position change and velocity of motion in depth [20]. A third study investigated time to contact for motion in depth with monocular and binocular cues [21]. Here, it was found that participants overestimated time to contact, suggesting they did not utilize the acceleration present in their stimulus.

Very few studies have compared motion perception for constant-world and constant-retinal motion. Adding self-acceleration to optic flow stimuli decreases the length of time before participants begin to feel vection, the feeling of self-motion, and increases the perceived speed of self-motion compared to when a constant velocity stimulus is used [22]. Additionally, if an accelerating stimulus, rather than a decelerating stimulus, is presented and followed by a speed discrimination task, speed discrimination thresholds are reduced, suggesting that if there is a “context” of acceleration, speed discrimination may be easier [23]. However, as described above, acceleration appears not to be useful when judging time to contact [21]. Thus, it is not clear whether the visual system is most sensitive to one or the other type of motion.

In the experiment discussed here, we were interested in how the speed of motion in depth is processed. However, measuring speed discrimination is notoriously difficult because in most paradigms observers can use duration or distance rather than specifically responding to speed [24]. We therefore chose a speed discrimination task that observers could not perform by using distance or duration alone, namely a speed change discrimination task [13,25,26]. In this task, participants viewed a standard interval which travelled at either a constant speed in the world or on the retina, and an interval that travelled equal amounts slower and then faster than the speed in the standard interval. The average speed over the course of the speed change interval was the same as in the standard interval, and the distance travelled and duration were the same as in the standard interval.

For the speed change discrimination task we chose two main 3D motion stimuli types. Our *World* stimulus contained binocular and looming cues and travelled at constant world speeds (accelerating retinal speed). Our *Retina* stimulus contained binocular and size-change cues and travelled at constant retinal speeds. As well as these, we used two control stimuli. The *World Control* stimulus moved with accelerating retinal speed and contained lateral motion and looming information only. The *Retina Control* stimulus moved the same speed and distance on the retina as the *Retina* stimulus, but contained no cues to motion in depth and moved laterally. Using these stimuli, our aim was to investigate whether the visual system was more or less sensitive to speed changes in motion in depth for constant Real World or Retinal motion using a speed change discrimination task.

## Methods

### Participants

11 participants (8 female, 3 male) were recruited for this experiment. The number of participants to recruit was decided in advance of data collection. This study can be considered a classic small-N design typical of visual psychophysics using many measurements per participant; the advantages for using these designs has been discussed in some detail [27]. All participants had normal or corrected-to-normal vision and were required to have a stereoacuity of at least 120 arcseconds as measured by a TNO test (16^th^ edition). One participant was rejected for having a higher stereoacuity threshold, and another was rejected for failing to reach 80% correct responses in the highest speed change level of two conditions, leaving 9 participants (7 female, 2 male) to complete the study. Prior to the experiment start, all participants gave written informed consent. This consent procedure and all other procedures were approved by the St. Andrews University Teaching and Research Ethics Committee (UTREC; Approval code: PS11904) and adhered to the tenets of the Declaration of Helsinki.

### Materials

Stimuli were presented using a Mac Pro on an Iiyama MM904UTA Vision Master Pro 455 cathode ray tube screen with a resolution of 1280x1024 and a refresh rate of 85Hz. The screen was luminance calibrated using a Cambridge Research Systems ColorCal MK II colorimeter and size calibrated by manually measuring the size of a square of a set number of pixels on the screen to obtain an accurate pixel per centimeter conversion. The screen was viewed through a four-mirror stereoscope from a distance of 97cm (including the distances between the mirrors of the stereoscope).

### Stimulus design

All stimuli were generated using MATLAB R2014b (The MathWorks Inc., 2014) with Psychtoolbox-3 [28–30]. All code used to generate the stimuli and run the experiment, has been deposited in an open access repository and available via https://osf.io/7mg63/. Two different stimulus types were used in these experiments. Both stimuli were presented on a grey screen with a luminance of 29.9 cd/m^2^. The first consisted of a drifting grating subtending 4 degrees of visual angle with a spatial frequency of 1 cycle per degree moving laterally from left to right at a constant retinal speed. This drifting grating stimulus was used exclusively in 2 training blocks.

**Fig 2:**
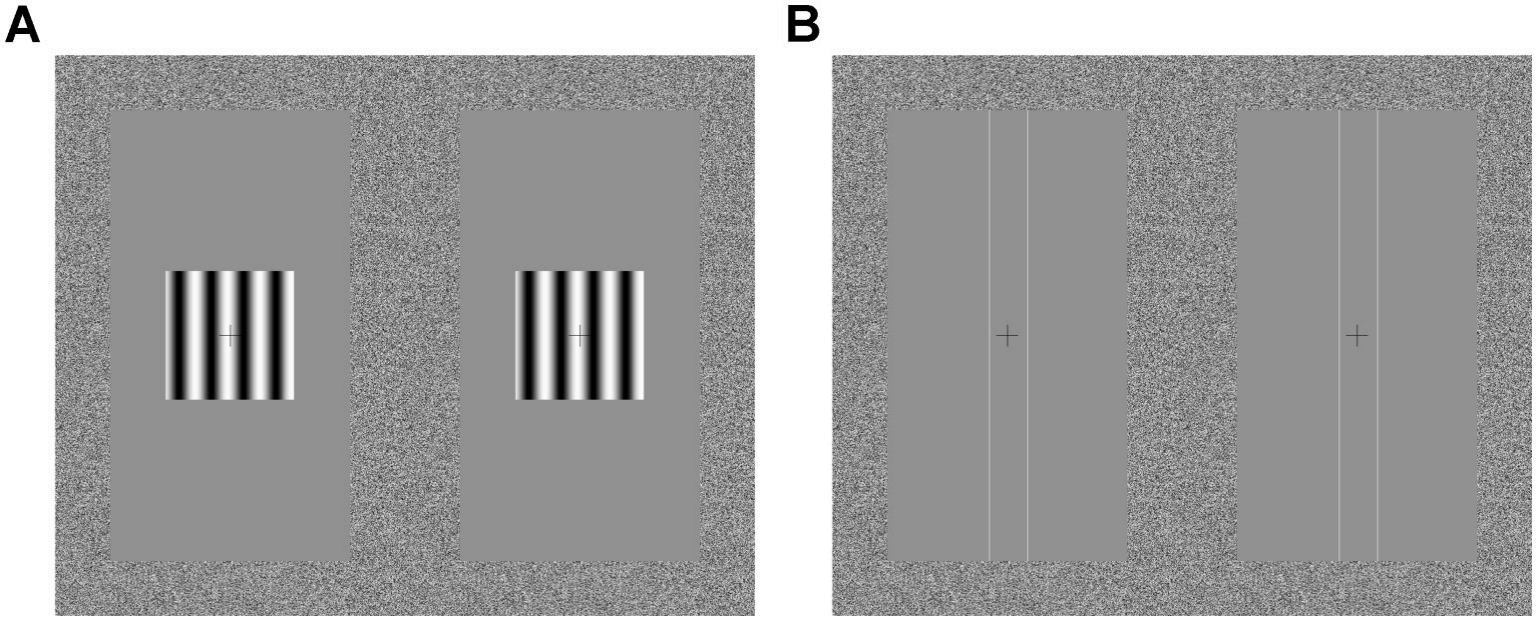
The stimuli presented to participants. (A) Shows the drifting grating stimulus used only for training purposes. (B) Shows the moving line stimulus. The left side of the image is viewed only by the left eye. The right side of the image is viewed only by the right eye. The two halves of both stimuli are fused into a single image by the mirror stereoscope.

The other stimulus was a pair of vertical white lines with a luminance of 59.9 cd/m^2^ that could either move laterally or in depth, with constant world or constant retinal speed. Stimuli moving towards the observer contained both binocular (from changing binocular disparity and interocular velocity difference) and looming (from the increasing separation between the lines) cues to motion in depth. The lines themselves did not change size and remained a constant 0.95 arcmin wide on the screen. These lines were 13.0 degrees tall. An aperture frame of close-to uniformly distributed luminance noise (individual pixels given randomly chosen grey levels) 1.58 degrees wide with a minimum luminance of 0.09 cd/m^2^ and a maximum luminance of 59.9 cd/m^2^ was also presented to aid fixation in the plane of the screen. The vertical line stimulus spanned the full height of the monitor that was within the aperture. Stimuli were presented with a central white fixation cross (37.9 arcmin tall, 37.9 arcmin wide) with a luminance of 59.9 cd/m^2^. A 56.9 by 56.9 arcmin white box with a luminance of 59.9 cd/m^2^ appeared around the fixation cross when participants were to give their responses. The moving line stimulus was used in 1 training condition and 8 experimental conditions. The moving line stimulus, and the drifting grating stimulus used for training, are shown in Fig 2. Examples of the moving line stimulus are shown in S1 and S2 Figs.

**Fig 3:**
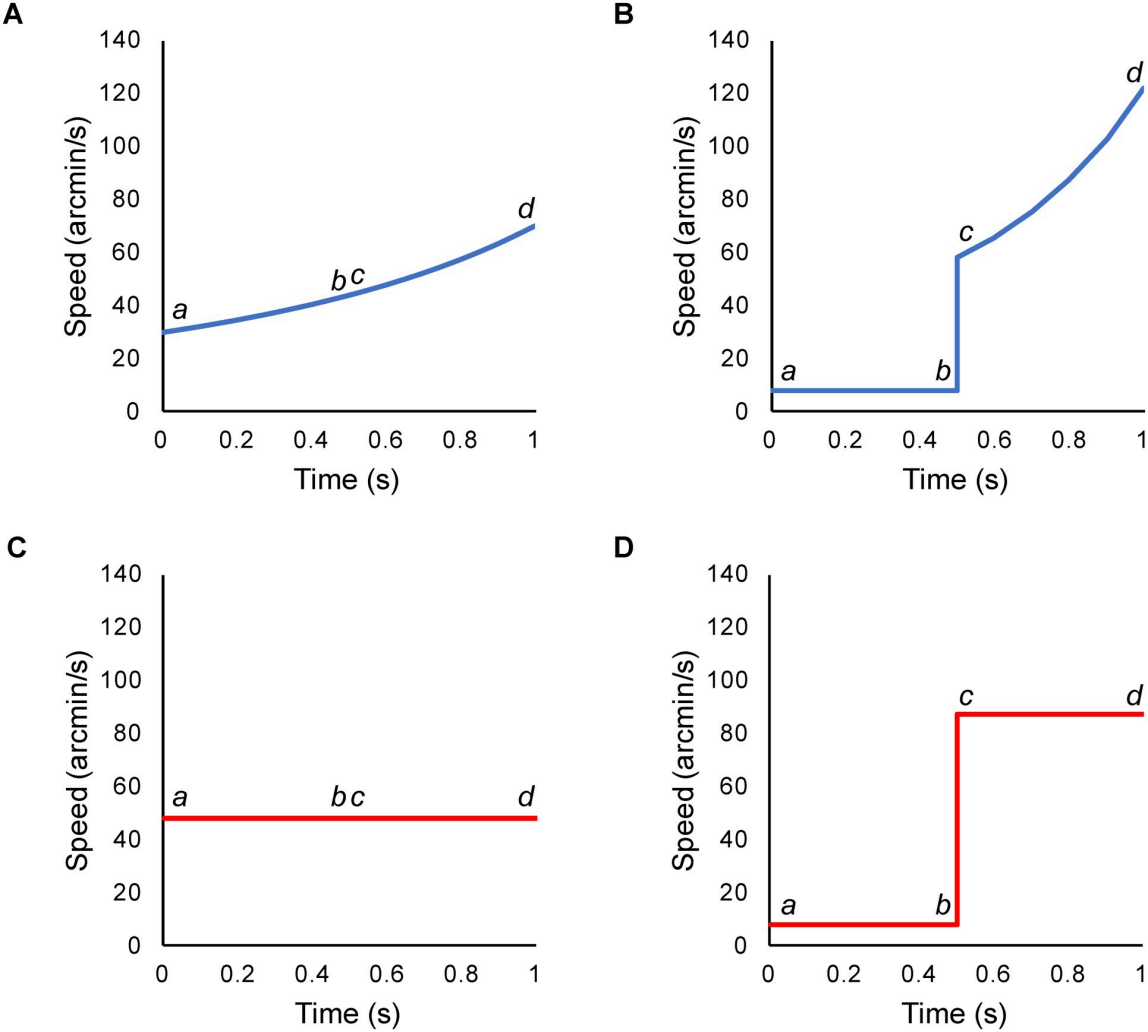
The structure of speed change intervals represented as a speed-time graph for retinal speed. (A) shows a ‘standard’ speed change interval (no change in speed) for the *World* and *World Control* conditions whilst (B) shows a ‘variable’ speed change interval (instantaneous speed changes during the trial) for the *World* and *World Control* conditions. (C) shows a standard speed change interval for the *Retina* and *Retina Control* conditions and (D) represents a maximum speed change interval in the *Retina* and *Retina Control* conditions. *a* represents the speed at the very beginning of the interval, whilst *b* represents the speed immediately before the step change in speed. *c* the speed immediately after the speed change, and *d* represents the speed at the very end of the interval.

Each experimental trial contained two intervals. One was labeled as the ‘standard’ speed change interval, where the lines moved at a constant speed. The other was the ‘variable’ interval, which contained an instantaneous speed change during the trial. Four stimulus types and associated experimental conditions were designed:

(I) *World*: constant world speed of motion in depth. Here each eye’s view contained lines moving with accelerating motion on the retina. Fig 3A shows how the speed changed on the retina during the standard interval, and Fig 3B shows an example of retinal speed during a variable interval. Both binocular and looming cues to motion in depth were present.
(II) *World Control*: lateral motion consistent with one eye’s view from the “World” stimulus, but presented to both eyes, so containing looming but not binocular cues to motion in depth (Fig 2A and 2B show retinal motion for different *World Control* intervals).
(III) *Retina*: constant retinal speed of motion in depth. Here, each eye’s view contained lines moving at a constant retinal speed, consistent with decelerating motion in the world (retinal motion for a standard speed change interval is shown in Fig 3C and a variable speed change interval in Fig 3D). The lines moved apart from each other at a constant retinal speed.
(IV) *Retina Control*: lateral motion with speeds consistent with one eye’s view from the “Retina” stimulus, but presented to both eyes. There were no binocular cues to motion in depth and no looming or size-change cues (Fig 2C and 2D shows the retinal motion).

#### World and World Control stimuli

For the *World* stimuli, we accurately rendered the retinal view for a real object moving in depth at a constant speed. Calculating the correct stimulus location to render was done using the following equations:

$$\theta ={tan}^{-1}\left(\frac{x_{w}}{z_{w}}\right)$$ (1)

$$x_{s}=Dtan\theta$$ (2)

**Fig 4:**
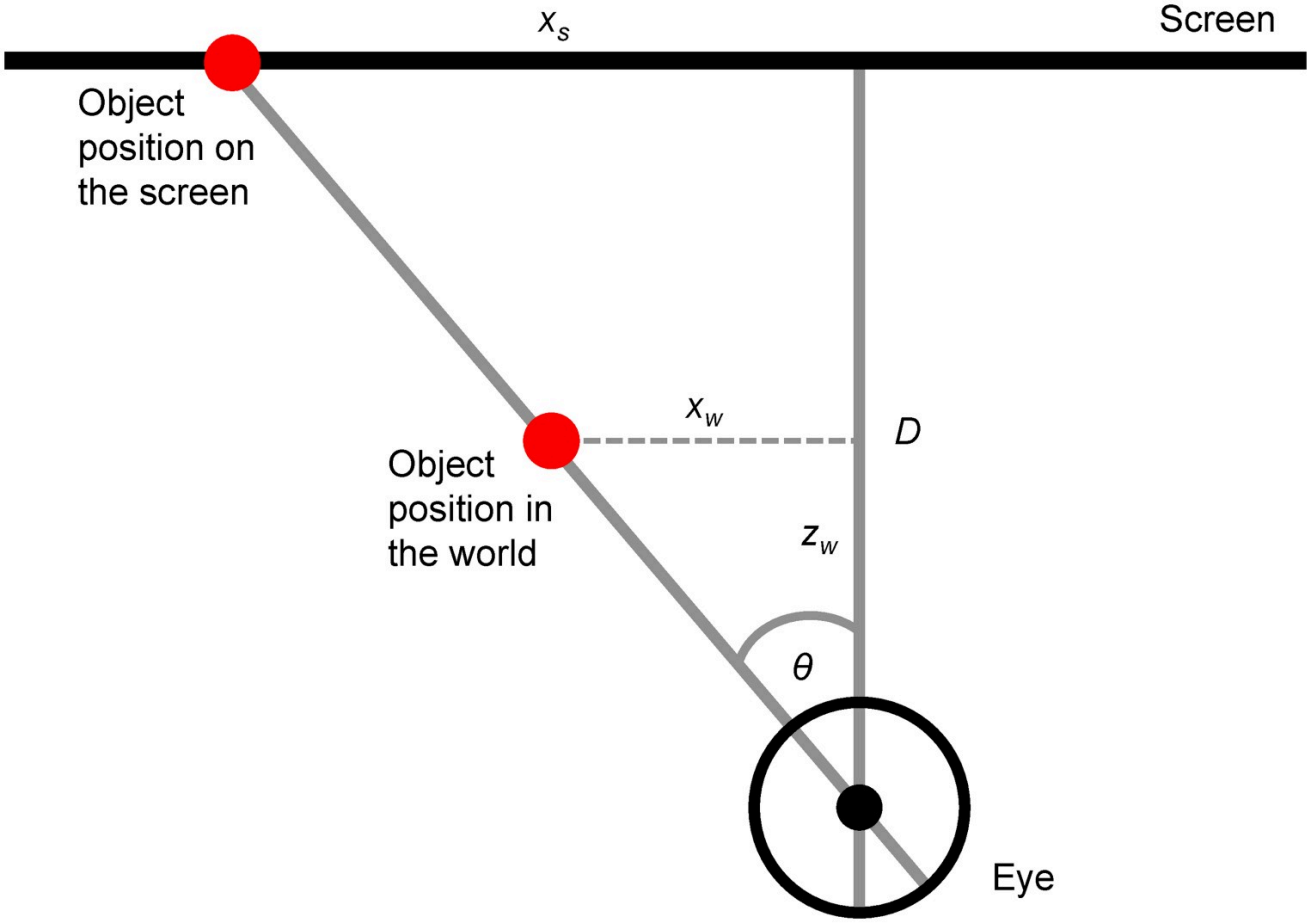
A visual representation of the values used to calculate the correct position for a 3D object to appear on a 2D screen.

Where *θ* is the visual angle, *x*_*w*_ is the position of the object along the x axis of the real world, *z*_*w*_ is the position of the object along the z axis of the real world, *x*_*s*_ is the position of the object on the screen and *D* is the viewing distance (see Fig 4). Eq 1 converts world positions into a retinal angle. This retinal angle is then used in Eq 2 to find the position the object should appear at on the screen. For finding the correct stimulus location on the screen, Eqs 1 and 2 simplify to Eq 3:

$$x_{s}=D(\frac{x_{w}}{z_{w}})$$ (3)

As the retinal position is defined by the arctangent (see Eq 1), and the speed and acceleration are the derivative and second derivative of position respectively, this means the speed and acceleration of motion in depth are not constant. Instead, speed and acceleration increase as the stimulus approaches the retina, as is the case when viewing a real approaching object in the world. For the *World Control* stimulus, we took the left monocular half image from the *World* stimulus and displayed it to both eyes, thus the lines move from left to right but contain looming information. Like the *World* stimulus, the *World Control* stimulus contained accelerating retinal speed, as would be present if an object was approaching the observer. However, because the relationship between world and retinal speed is close to linear for 2D motion except at large eccentricities, and because the *World Control* accelerates as if the stimulus is moving towards the observer, the *World Control* stimulus is not representative of a 2D object moving at a constant world speed.

#### Retina and Retina Control stimuli

For the *Retina* stimulus, each eye’s view was delivered at constant retinal speed, but in opposite directions, specifying binocular motion in depth. This is consistent with decelerating motion in the world. The stimulus contained size change information similar to the looming information in the *World* stimulus, but with size change at a constant retinal speed. The size-change cue was not identical to looming as looming by definition involves non-constant retinal speeds. The *Retina Control* stimulus consisted of lateral motion at a constant retinal speed, but with no looming information present. The speeds used in these two conditions were calculated by taking the mean of the speeds presented to the observer in the *World* conditions–see below for details.

### Procedure

**Fig 5:**
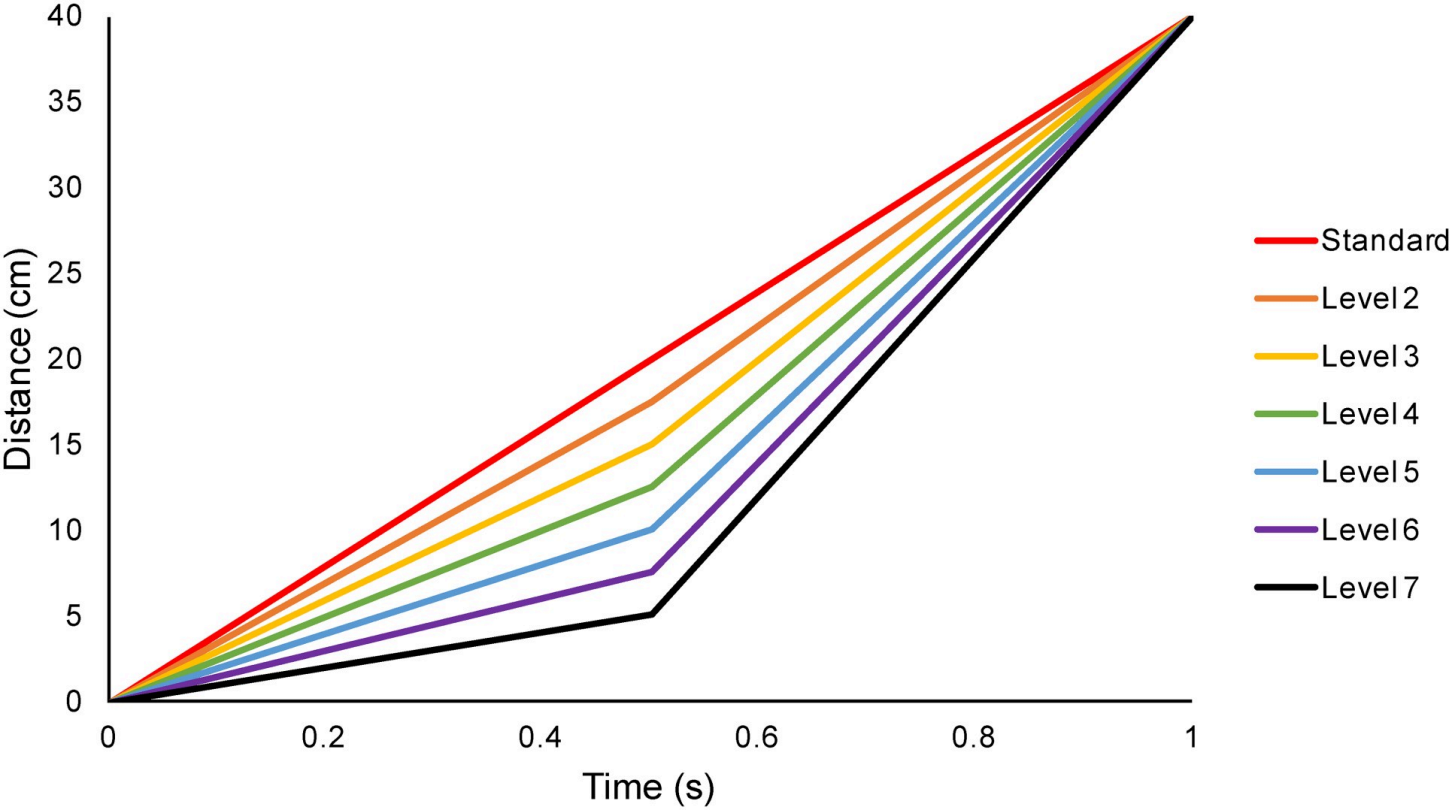
A distance-time graph of the different levels used in a speed change discrimination experiment. Demonstrates how the stimulus for each level of speed change discrimination stimuli travelled over the same total distance in the same total duration, in terms of the distance travelled in the real world.

A 2-interval forced choice task was used. The participant’s goal was to identify the interval that contained a step change in speed (like those in Fig 2B and 2D), compared to an interval that did not have this step change (like those in Fig 2A and 2C). Using a speed *change* discrimination task such as this, rather than a traditional speed discrimination task, allows us to control against the use of both distance and duration cues in speed perception. This was accomplished by carefully choosing the speeds used in the changing stimulus so that the total duration and total distance travelled was identical in the standard and test intervals (see Fig 5). Thus, the first half of the stimulus was slower than the standard and the second half of the stimulus was faster than the standard.

In each interval, a static image of the stimulus appeared for 250ms before moving for 1 second. If a speed change were to occur in the interval it would always happen after 500ms of motion. The stimuli in each condition were presented using a 7-level method of constant stimuli design. Each condition was divided into three blocks with 10 trials per level, giving 70 trials per block and 210 per condition. Blocks were presented in a random order. No feedback was given during the main experiment, we aimed to avoid training participants from using something other than the speeds present to complete the task. In each trial participants heard one beep immediately before the first interval, and two beeps immediately before the second interval.

#### Training blocks

The three training blocks were always presented in the same order. The first training block consisted of the drifting grating stimulus moving at a constant retinal speed and presented with audio feedback. If the participant correctly picked which grating changed speed they would hear a high-pitched beep, and if they incorrectly picked, they would hear a low-pitched beep. The second training block was visually identical to the first but had no audio feedback. The third block used the *Retinal Control* stimulus with the two moving lines and no feedback. In each training block participants viewed 30 trials with an “easy” speed change and 30 trials with a “hard” speed change, giving a total of 180 training trials. In the standard intervals of these training conditions, the stimuli moved at 283.4 arcmin/s on the retina. In the “hard” test intervals, the stimulus changed from moving at 212.6 arcmin/s to 354.1 arcmin/s. In the “easy” test intervals, the stimulus initially moved at 70.9 arcmin/s before jumping to 495.3 arcmin/s. Participants were required to obtain correct performance on at least 50 out of a total of 60 trials in the final training block to proceed to the main experiment. No participants were excluded based on their performance in the training blocks.

#### Main experiment

For the main experiment, we used 8 experimental conditions. Our conditions were named in accordance with our four stimuli types listed above, each with “fast” and “slow” speeds. “Fast” speeds were based around a standard interval of approximately 40cm/s in the world while “slow” speeds were based around a standard interval of approximately 20cm/s in the world. We will refer to each condition using a short-hand term, for example, *World-Fast* is our fast-speed condition replicating 3D motion in the real world by using fast accelerating retinal speeds, and *Retina Control-Slow* is the control version of the 3D Retina Slow condition. As a result, the stimuli listed above gave rise to the following 8 experimental conditions: *World-Fast*, *World-Slow; World Control-Fast*, *World Control-Slow; Retina-Fast*, *Retina-Slow*, *Retina Control-Fast*, and *Retina Control-Slow*. A summary of the speeds in arcmin/s used in the Fast conditions is shown in Table 1, whilst a summary of the speeds in arcmin/s used in the Slow conditions is shown in Table 2. We include details for the standard interval, and for the maximum change test interval (level 7 in Fig 5).

**Table 1:** The speed(s) presented in the standard and maximum speed change level for each of the fast speed conditions before and after the speed change.

Condition and level	Speed(s) before change (arcmin/s)	Speed(s) after change (arcmin/s)
World		
Fast standard	30.1–43.8	43.9–69.5
Fast maximum change	7.5–8.2	57.6–121.4
Control-Fast standard	30.1–43.8	43.9–69.5
Control-Fast maximum change	7.5–8.2	57.6–121.4
Retina		
Fast standard	47.6	47.6
Fast maximum change	7.6	87.4
Control-Fast standard	47.6	47.6
Control-Fast maximum change	7.7	87.5

**Table 2:** The speed(s) presented in the standard and maximum speed change level for each of the slow speed conditions before and after the speed change.

Condition and level	Speed(s) before change (arcmin/s)	Speed(s) after change (arcmin/s)
World		
Slow standard	18.0–21.9	21.9–27.2
Slow maximum change	4.5–4.7	33.1–47.6
Control-Slow standard	18.0–21.9	21.9–27.2
Control-Slow maximum change	4.5–4.7	33.1–47.6
Retina		
Slow standard	22.3	22.3
Slow maximum change	4.6	40.0
Control-Slow standard	22.3	22.3
Control-Slow maximum change	4.6	40.1

The speeds in arcmin/s for the *World-Fast* condition standard level correspond to a constant world speed of 40 cm/s in depth. The speeds for the maximum change level from this condition correspond to world speeds of 10 cm/s in depth before the change and 70 cm/s in depth after the change. The world speeds for each level of this condition are shown in Table 3. For the *World-Slow* condition, the standard level speed corresponds to a world speed of 20 cm/s and the maximum change level speeds correspond to a speed of 5 cm/s before the speed change and 35 cm/s after the speed change. The world speeds for the levels of the *World-Slow* condition are shown in Table 4. S1 and S2 Figs demonstrate a speed change interval and a standard interval respectively from the *World-Fast* condition.

**Table 3:** The constant world speeds for each level of the World-Fast condition.

Level	Speed before change (cm/s)	Speed after change (cm/s)
1 (standard)	40	40
2	35	45
3	30	50
4	25	55
5	20	60
6	15	65
7 (maximum change)	10	70

**Table 4:** The constant world speeds for each level of the World-Slow condition.

Level	Speed before change (cm/s)	Speed after change (cm/s)
1 (standard)	20	20
2	17.5	22.5
3	15	25
4	12.5	27.5
5	10	30
6	7.5	32.5
7 (maximum change)	5	35

### Analysis

Percent correct responses were recorded as a function of proportion speed change at the changepoint, which was calculated using Eq 4:

$$\frac{c-b}{c}$$ (4)

where *c* is the speed immediately after the speed change and *b* is the speed immediately before the speed change, as shown on the graphs in Fig 3. Percent correct responses were analyzed in this way to avoid biasing the results due to the increased speed in the *Fast* conditions, or due to the higher end speeds of the *World* and *World Control* conditions compared to the *Retina* and *Retina Control* conditions. From participant responses, 75% correct thresholds for determining which interval contained the speed change were found for each condition by fitting cumulative normal psychometric functions using MATLAB R2014b (The MathWorks Inc., 2014) with the Palamedes toolbox [31]. A fixed guess rate of 0.5 and a fixed lapse rate of 0 was used for all psychometric function fits. Psychometric functions for all 8 conditions from a representative participant can be found in S3 Fig.

### Statistics

Following psychometric analysis, JASP Version 0.9.1 (JASP Team, 2018) was used to conduct multiple 2-way repeated measures ANOVAs on our data. The first ANOVA involved a comparison of the *World* and *Retina* thresholds, as well as a comparison of the corresponding *Slow* and *Fast* thresholds. The second ANOVA compared the *World* and *World Control* thresholds along with the corresponding *Slow* and *Fast* thresholds. The final ANOVA compared the *Retina* and *Retina Control* thresholds and the corresponding *Fast* and *Slow* thresholds. We performed 3 2-way repeated measures ANOVAs rather than a single 3-way repeated measures ANOVA because we wanted to test something different with each comparison. With the comparison between the *World* and *Retina* conditions we wanted to compare speed change discrimination thresholds for constant world and constant retinal speeds. For our *World* and *World Control* conditions, we were interested in what effect removing binocular cues to motion in depth would have on the speed change discrimination thresholds. For our *Retina* and *Retina Control* conditions, we wanted to observe the effect of removing all cues to motion in depth on the speed change discrimination thresholds. It would not be possible to make these comparisons with a 3-way repeated measures ANOVA. In order to be able to determine if the null or alternate hypothesis was more likely, Bayesian repeated measures ANOVAs were also conducted. These provide different models to explore which differences within the ANOVA factors explain the data. The prior used was a Cauchy distribution, centered on zero, with a width of 0.5, which is the default prior for Bayesian repeated measures ANOVAs in JASP Version 0.9.1. For a review of the rationale for using Bayesian statistics in comparison to frequentist statistics see [32]. We are reporting Bayes Factors in two ways. BF_10_ values indicate the ratio of the likelihood of the alternative compared to the null. BF_01_ values give the ratio of the likelihood of the null compared to the alternate. These quantities are the inverse of each other.

The data collected in this experiment and the code written to analyze the data has been deposited in an open access repository and is available at https://osf.io/7mg63/.

## Results

**Fig 6:**
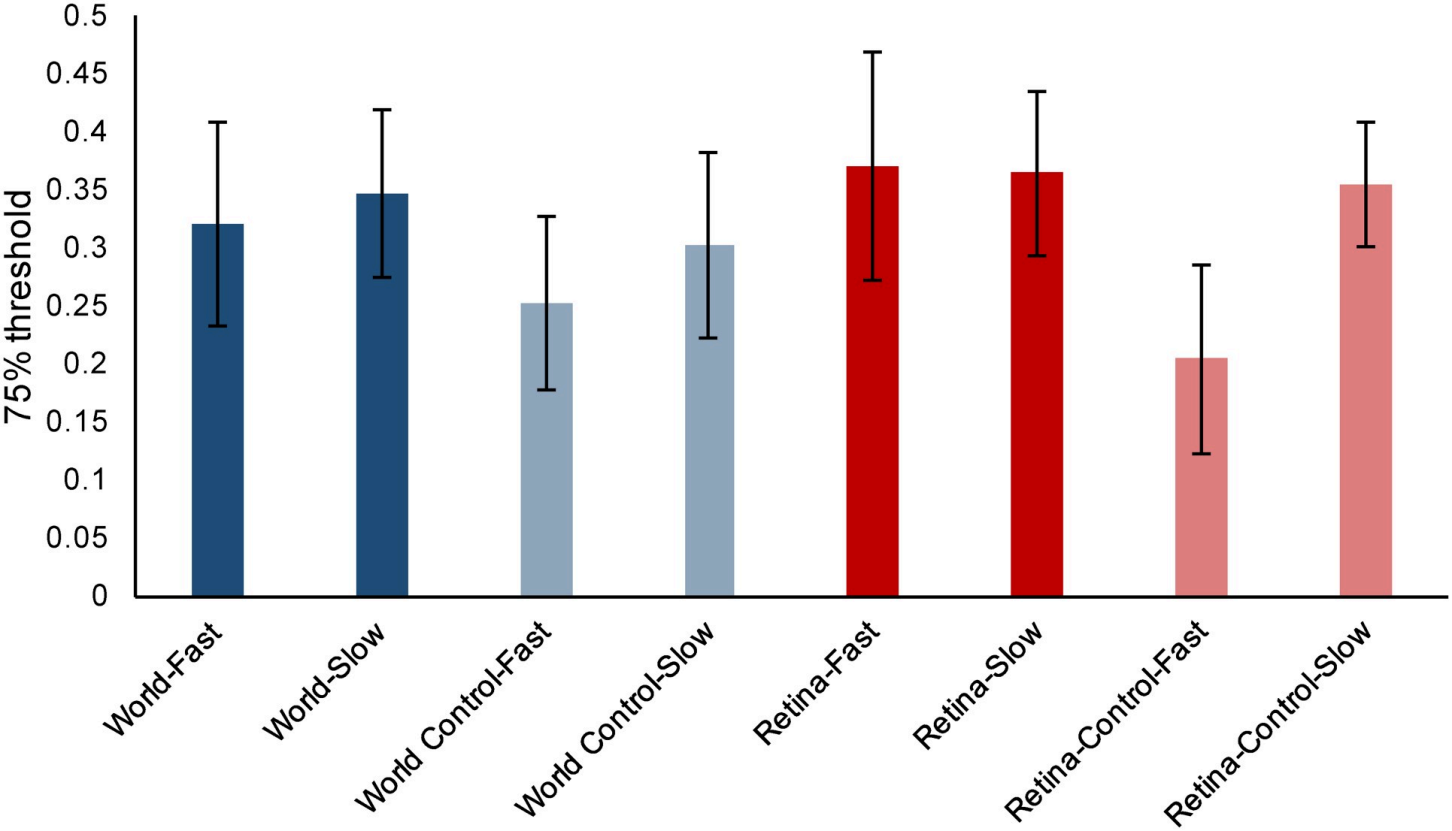
75% proportion speed change thresholds. Blue bars represent World conditions while red bars represent Retina conditions. Lighter coloring indicates a control condition. Error bars are between-subject confidence intervals.

A summary of the proportion speed change thresholds for each of our 8 conditions is shown in Fig 6. The results of this experiment are then considered in terms of the comparison between our *World* and *Retina* conditions, our *World* and *World Control* conditions and our *Retina* and *Retina Control* conditions.

### World versus Retina

The 75% thresholds across observers, described in terms of the proportion speed change at the changepoint, were very similar across all four *World* and *Retina* conditions (see Fig 6). We found no significant difference between the *World* and *Retina* conditions (F(1,8) = 0.959, p = 0.356, η^2^ = 0.107) or the *Fast* and *Slow* conditions (F(1,8) = 0.324, p = 0.585, η^2^ = 0.039). We also found no interaction effect between our *World* vs. *Retina* and *Fast* vs. *Slow* comparisons (F(1,8) = 0.461, p = 0.516, η^2^ = 0.054).

We also conducted Bayesian statistical analyses to obtain Bayes Factors for the *World* vs. *Retina* and corresponding *Fast* vs. *Slow* data. These allow us to investigate more carefully the likelihood of the data being consistent with the alternative hypothesis (difference between thresholds across conditions) or the null hypothesis (no difference between thresholds across conditions). The Bayesian repeated measures ANOVA conducted here provides Bayes Factors for 4 different models which may explain the data: (1) A *World vs*. *Retina* model, which assumes that only the difference between the *World* and *Retina* conditions explains the data. (2) A *Fast vs*. *Slow* model, which assumes that only the differences between the *Fast* and *Slow* conditions explain the data. (3) A *World vs*. *Retina* and *Fast vs*. *Slow* model, which assumes that the linear combination of differences between *World* and *Retina* and *Fast* and *Slow* explain this data. (4) A *World vs*. *Retina*, *Fast vs*. *Slow* and interaction model, which assumes that the data can be explained by the differences between the *World* and *Retina* conditions, the *Fast* and *Slow* conditions and an additional interaction between these factors. A Bayes factor of 3 or larger is considered substantial evidence that a set of results support either the null (if using a BF_01_ value that is weighted toward the null) or alternative (if using a BF_10_ value that is weighted towards the alternative) hypothesis, while a Bayes factor between 1 and 3 only indicates weak or anecdotal evidence in support of a hypothesis [33–35].

A Bayes Factor (BF_01_) of 1.596 was found for the *World* vs. *Retina* model, indicating anecdotal support for the null hypothesis and that these results are 1.596 times more likely under the null hypothesis. A Bayes Factor (BF_01_) of 2.981 was found for the *Fast* vs. *Slow* model, indicating these findings are 2.981 times more likely under the case of the null hypothesis, and anecdotal support for the null hypothesis. A Bayes Factor (BF_01_) of 4.802 was found for the *World vs*. *Retina* and *Fast vs*. *Slow* model, indicating substantial support for the null hypothesis. A Bayes Factor (BF_01_) of 10.582 was found for the *World vs*. *Retina*, *Fast vs*. *Slow* and interaction model, which indicates strong evidence for the null hypothesis [33,35]. For each tested model it is more likely to observe the data if the null hypothesis was true than if the alternate hypothesis was true. These Bayes factors suggest that either the visual system is as sensitive to changes in constant world speed or constant retinal speed or any difference in sensitivity is small.

### World versus World Control

75% thresholds were higher for the *World* conditions compared to the *World Control* conditions (see Fig 6). *World Control* stimuli delivered significantly lower thresholds than the *World* condition(F(1,8) = 8.116, p = 0.022, η^2^ = 0.504). There was no significant difference between the *Fast* and *Slow* conditions (F(1,8) = 4.404, p = 0.069, η^2^ = 0.355), and no significant interaction between the *World* vs. *World Control* comparison and the *Fast* vs. *Slow* comparison (F(1,8) = 1.036, p = 0.338, η^2^ = 0.115).

Similar to for the *World vs*. *Retina* data, the Bayesian repeated measures ANOVA used here provided 4 models that could explain the *World vs*. *World Control* data. For the *World* vs. *World Control* model, a Bayes Factor (BF_10_) of 9.229 was found. This is considered substantial evidence in favor of the alternative hypothesis that the data is explained by a difference between the *World* and *World Control* conditions [33–35]. It also suggests that this data is 9.229 times more likely to occur if the alternative hypothesis is true. A Bayes Factor (BF_10_) of 1.227 was found for the *Fast* vs. *Slow* model, indicating ‘anecdotal support’ for the alternative hypothesis that there was a difference between these *Fast* and *Slow* conditions [34]. A Bayes Factor (BF_10_) of 18.472 was found for the model that linearly combines *World vs*. *World Control* and *Fast vs*. *Slow*, indicating strong support for the alternative hypothesis [33,35]. A Bayes Factor (BF_10_) of 8.647 was found for the *World vs*. *World Control*, *Fast vs*. *Slow* and interaction model, suggesting substantial evidence for the alternative hypothesis. These findings indicate that the addition of binocular motion in depth information may make the speed change discrimination task more difficult. In all cases the alternate model is more likely than the null model. The model using a linear combination of *World vs*. *World Control* and *Fast vs*. *Slow* factors has the highest Bayes Factor, making it the most likely model given the data. In addition, the Bayes Factor decreases when the interaction term is added to this model indicating that there is evidence against including the interaction term.

### Retina versus Retina Control

The *Retina Control-Fast* thresholds were lower than for the other *Retina* and *Retina Control* conditions (see Fig 6). We found a significant difference between the *Retina* and *Retina Control* conditions (F(1,8) = 5.484, p = 0.047, η^2^ = 0.407) and the corresponding *Fast* and *Slow* conditions (F(1,8) = 6.183, p = 0.038, η^2^ = 0.436). We additionally found a significant interaction effect between the *Retina* vs. *Retina Control* comparison and the *Fast* vs *Slow* comparison (F(1,8) = 46.360, p < 0.001, η^2^ = 0.853).

However, a Bayes Factor (BF_10_) of 1.532 for a *Fast* vs. *Slow* model from the Bayesian repeated measures ANOVA suggests only anecdotal support for the alternative hypothesis that there is a difference between the *Fast* and *Slow* conditions [34]. The Bayes Factor (BF_10_) for the *Retina* vs. *Retina Control* model was 3.885, suggesting substantial support for the alternative hypothesis that the data is explained by differences between the Retina and Retina control conditions [33–35]. A Bayes Factor (BF_10_) of 8.370 was also found for the *Retina vs*. *Retina Control* and *Fast vs*. *Slow* model, indicating substantial support for the alternative hypothesis. A Bayes Factor (BF_10_) of 40.682 was found for the *Retina vs*. *Retina Control*, *Fast vs*. *Slow* model and interaction model, indicating very strong support for the alternative hypothesis that this data is explained by the differences between the Retina and Retina Control conditions, the Fast and Slow conditions and the interaction between these two factors [33,35]. This suggests that removing both monocular and binocular motion in depth information from stimuli with a constant retinal speed made the speed change discrimination task easier.

## Discussion

We hypothesized that the visual system might be more sensitive to changes in constant world speeds, which accelerate on the retina, than to changes in constant retinal speeds. Our results show no significant difference between constant world speed and constant retinal speed conditions, as demonstrated by our *World* vs. *Retina* comparison. Bayes factors provide anecdotal evidence that there is no difference between the *World* and *Retina* speed conditions. However, these results suggest that when 3D motion cues are removed, thresholds are lower. Mixed evidence regarding whether it easier to do the task when the conditions contained fast speeds was also found. Below we discuss how our results compare with literature on the perception and use of acceleration in visual stimuli, speed discrimination at different speeds and comparisons between 2D and 3D motion perception. We also discuss the nature of speed change discrimination in more detail, and speculate about why speed change discrimination thresholds are high.

### Perception of acceleration

Although motion with constant speed in the world has greater ecological validity, there is evidence that the acceleration in the more ecologically valid stimuli is difficult to perceive. It has been demonstrated that constant acceleration is difficult to perceive and discriminate, particularly in comparison to speed discrimination [36–42].

Whilst there appears to be a consensus on the difficulty of acceleration discrimination, estimates of how large the speed change needs to be for the acceleration to be visible varies greatly between studies, anywhere from a 25% to 3 times increase in speed [36,37,39,40,43,44]. It is also important to note that these studies were looking at acceleration discrimination, with the majority of using constant acceleration in their stimuli. This is different to the acceleration present on the retina when we view objects moving towards us in the world, and in our *World* stimuli, which is non-constant. For example, with a task where participants had to report whether a ball was accelerating, decelerating or moving at a constant speed, Schmerler [36] reported that for acceleration to be detected on 50% of occasions, the end speed needed to be 2.3 to 3.2 times different from the initial speed. Meanwhile, Gottsdanker et al. [37] used a task where an accelerating or decelerating speed had to be discriminated from a stimulus moving with a constant velocity, and found a range of 75% threshold values of percentage speed change, from 26% to 157%, that varied according to presentation time and mean velocity. Others have also reported thresholds of around a 20–25% change in speed for acceleration discrimination [43,44]. Even the values reported in these studies of a 20–25% change in speed as a threshold for acceleration discrimination is considerably higher than thresholds found for speed discrimination, which can be as small as a 5–7% difference in speed [10–12,15,18,19]. Despite the reported differences in acceleration thresholds, the literature suggests that acceleration discrimination is relatively difficult. This raises the question of whether accelerations like those present in our *World* and *World Control* conditions are perceived when they are available in stimuli. If they cannot, we may expect there to no difference between the *World* and *Retina* conditions, despite the increased ecological validity of the *World* conditions, as we have found here.

### Use of acceleration information

There is evidence to suggest that not only is acceleration difficult to discriminate, but it is also not used when it is present in time-to-contact tasks [21,44–48], or in manual interception tasks [49]. Using a task to discriminate an accelerating stimulus from a decelerating stimulus, Brouwer et al. [44] suggested that participants were likely comparing the start and end speeds of the stimuli rather than judging acceleration itself, concluding that people do not use acceleration during interception of approaching objects. There is evidence to suggest that we are better at intercepting or catching objects that have been accelerated by gravity, and that we may have an internal model for gravity [50–52]. However, it also appears that we anticipate gravitational acceleration, which may suggest again that instead of perceiving the acceleration itself, our improved ability to intercept gravity-accelerated objects may be due to experience with gravity [53].

To sum up, the majority of evidence appears to suggest that acceleration is likely rarely used by the visual system, and when we are forced to make discriminations about it, these are usually very difficult. We found no clear difference in speed change thresholds between constant world speed (acceleration on the retina) and constant retinal speed. This may suggest that the acceleration on the retina in our *World* conditions may not have been used by the visual system.

### Comparing lateral motion and motion in depth

We report a significant difference in the *World* vs. *World Control* and the *Retina* vs. *Retina Control* comparisons, with Bayes factors for both comparisons indicating substantial evidence that removing either binocular, or both binocular and monocular, cues to motion in depth from a stimulus made the task easier. These findings are consistent with a growing body of evidence that suggests several aspects of motion in depth are harder to perceive than their respective lateral motion equivalents [17,54–60]. The idea of stereomotion suppression was first proposed by Tyler [55,60], who found that sensitivity to depth movements was reduced compared to lateral movements of the same size on the retina, and reasoned that it must be a result of the processes involved in fusing the two eyes’ images. It has been suggested that motion in depth thresholds may be higher due to the early averaging of motion signals between the two eyes, which would give an average motion of close to zero for motion in depth, making it harder to perceive [56,57]. Importantly, stereomotion suppression has also been demonstrated in speed discrimination tasks [17]. Brooks et al. [17] found that for their random dot stimuli, speed discrimination thresholds for lateral motion were a factor of 1.8 lower than those for the respective motion in depth stimuli. Our results support these previous findings of stereomotion suppression for 3D motion and indicate that this effect also exists for speed change discrimination.

### Comparing faster and slower speeds

In our comparison between the *Retina* and *Retina Control Fast* and *Slow* conditions, we found that speed change discrimination was significantly easier with faster speeds. However, the Bayes factor indicated only anecdotal support for the hypothesis that there would be a difference between the slow and fast speeds. We additionally found there was no significant difference between the other *Fast* and *Slow* conditions.

Even the fast speeds used here were much slower than those typically used in the 2D motion literature. This is because large 3D motions translate into small motions on the retina, and because motion in depth experiments that include binocular disparity typically use slow retinal speeds to ensure that stimuli remain binocularly fused. However, there are several studies, for 2D and 3D motion, demonstrating that speed discrimination thresholds are worse at very slow speeds [3,10–12,15]. It has been reported for a motion in depth experiment using speeds of 0.2–0.3 deg/s that speed discrimination thresholds were in the range of 10–20% [3].

For 2D motion, speed discrimination thresholds are best within a range of 4–64 deg/s, and become progressively worse as motion is slower than 4 deg/s or faster than 64 deg/s [10,12]. McKee [11] demonstrated that below 2 deg/s thresholds grew towards 8–9%. For speeds as low as 0.5 deg/s, thresholds approach 18% [15]. The speeds used in our study were generally much slower than this (Tables 1 and 2). Based on the literature on speed discrimination, we would expect thresholds for all of the speeds used in our experiments to be sub-optimal, but we would still expect that our Fast speed change discrimination thresholds would be lower than our Slow speed change discrimination thresholds unless at the Fast speeds participants had already reached close to minimum performance abilities.

However, almost all the available literature has used a speed discrimination task. Speed change discrimination has barely been studied, and never, to our knowledge, for motion in depth. In the next section, we review our current understanding of speed change perception in more detail.

### The nature of speed change discrimination

Our is the first study to have explored speed change discrimination of motion in depth with binocular and monocular cues. Like other studies using a speed change task, thresholds were considerably higher than those reported previously for speed discrimination tasks. Such high thresholds have previously been observed for judgments of speed change discrimination of optic flow using random dot kinematogram stimuli [13,26]. For self-motion from optic flow, Monen et al. [26] found that dividing the two speeds into intervals separated in time cut the mean thresholds for their participants from around 49% to around 24%. Similarly, Snowden et al. [13] used random dot kinematograms moving in two dimensions and found that when the different speeds were in temporally-separate intervals, the speed change threshold could be as low as a 6%, but when the two speeds were presented in a single temporal interval and compared with another temporal interval with no speed change, the threshold rose to around 30%. It has also been found that speed discrimination thresholds can be up to three times worse when two speeds were presented with no time gap between the two stimuli [25].

There are several possible explanations for speed change discrimination having higher thresholds than classic speed discrimination. First, if temporal integration were needed to obtain a measure of speed, then speed change would be difficult to detect because the integration of speed information could average out the change. The notion of temporal integration being involved in making speed change discrimination tasks difficult is supported by Mateeff et al.’s [25] findings, because as the time between the speed presentations is reduced, thresholds increase. Others have also concluded that there is likely temporal integration of speeds when they are presented close together in time [61].

Second, spatial integration of speed may also play a role. It has been found that speed discrimination is more difficult when the task is to discriminate between two speeds for an object moving along one continuous trajectory, compared to having two stimuli travelling parallel to each other at different speeds [62]. Averaging over space would make it difficult to judge a speed change.

Third, speed change discrimination tasks prevent the use of both distance or duration cues. In contrast, in speed discrimination tasks it is very difficult to prevent participants from being able to use at least one of these cues [24]. It may be that many of the speed discrimination thresholds reported in the literature have not entirely eliminated all distance and duration cues and actually report combined thresholds for speed and distance or duration, rather than speed in isolation. Speed change discrimination thresholds may be poor because participants are unable to use duration or distance to help them to respond, and may struggle to discriminate between speeds in isolation. The cause of these elevated speed change discrimination thresholds is an interesting avenue for future research.

One further possible explanation is that our thresholds may be higher than those reported previously for speed discrimination as we used only naïve participants. With naïve observers, speed discrimination can also be poor: for example, Weber fractions around 0.15–0.35 for speed discrimination have been found using naïve observers [63]. These Weber fractions are closer to our mean proportion 75% thresholds of between 0.20 and 0.37.

## Conclusions

We aimed to compare speed change discrimination thresholds with a constant retinal speed, as frequently used in motion in depth studies, to those with a constant world speed, as we see when an object approaches us in the real world. We have found no evidence for a difference in speed change discrimination between these two types of stimuli. We have found results suggesting that thresholds for motion in depth are worse than those when motion in depth cues are removed. Additionally, we report speed change discrimination thresholds that are considerably higher than thresholds previously reported in speed discrimination tasks, consistent with other investigations using speed change discrimination.

## Supporting information

S1 Fig The moving line stimulus in a speed change interval of the *World-Fast* condition. (M4V)

S2 Fig The moving line stimulus in a standard interval of the *World-Fast* condition. (M4V)

S3 Fig Psychometric functions from a representative individual participant. Dots represent participant data and the line is the psychometric fit. (A) Shows the *World Fast* condition fit. (B) Is the fit for the *World Slow* condition. (C) Is the fit for the *World Control Fast* condition. (D) Shows the *World Control Slow* condition fit. (E) Shows the *Retina Fast* condition fit. (F) Shows the *Retina Slow* condition fit. (G) Is the fit for the *Retina Control Fast* condition. (H) Shows the *Retina Control Slow* condition fit.
(TIF)
