# Supplementary figures and images for: Speed change discrimination for motion in depth using constant world and retinal speeds
Source: PLoS One. 2019 Apr 3;14(4):e0214766. doi: 10.1371/journal.pone.0214766 (PMC6447190; doi:10.1371/journal.pone.0214766)

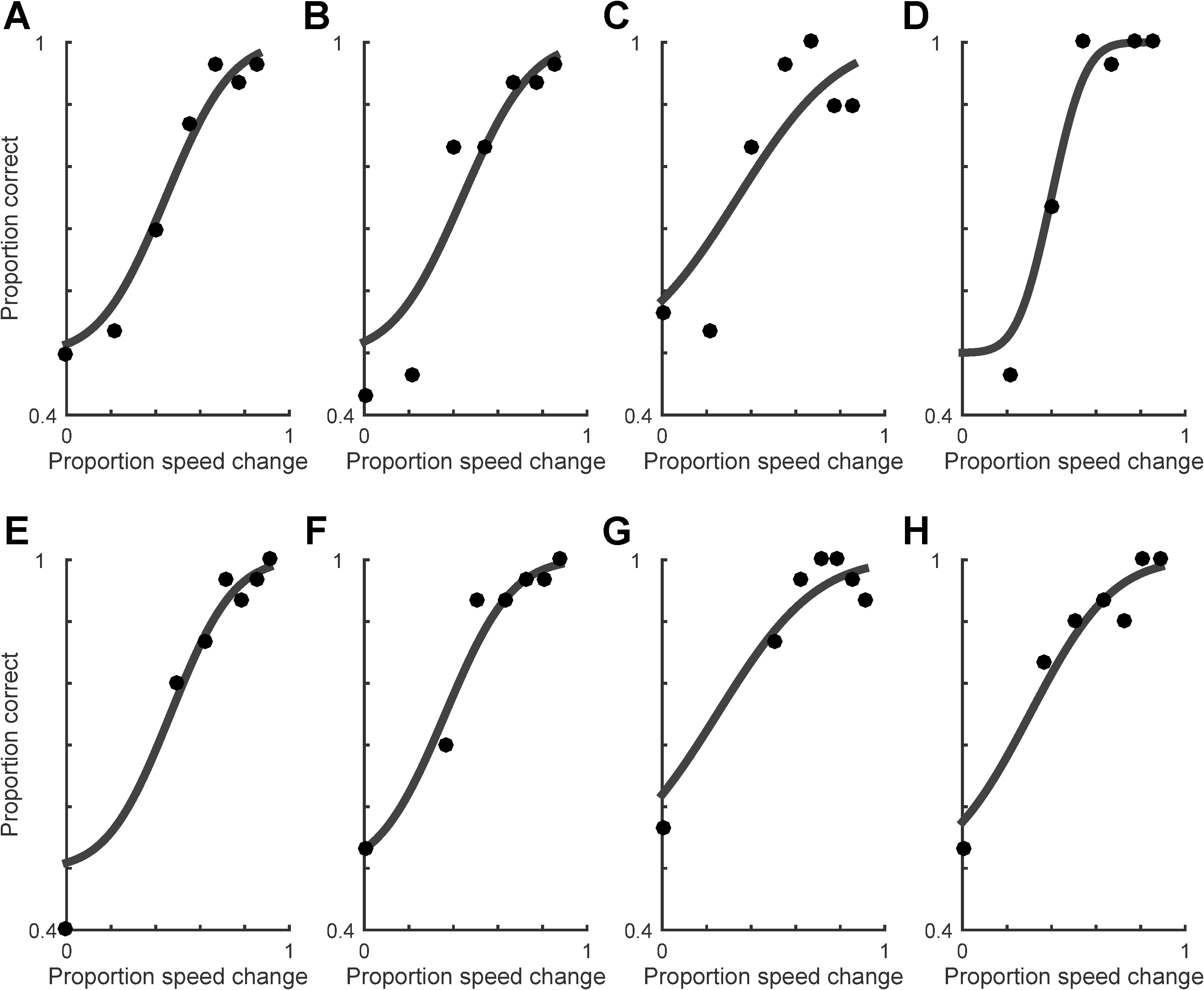

Supplement: S3 Fig — Dots represent participant data and the line is the psychometric fit. (A) Shows the World Fast condition fit. (B) Is the fit for the World Slow condition. (C) Is the fit for the World Control Fast condition. (D) Shows the World Control Slow condition fit. (E) Shows the Retina Fast condition fit. (F) Shows the Retina Slow condition fit. (G) Is the fit for the Retina Control Fast condition. (H) Shows the Retina Control Slow condition fit. (TIF) [file pone.0214766.s003.tif]
